# Editorial: Physical activity and exercise for the prevention and management of cardiovascular risk and cardiovascular disease

**DOI:** 10.3389/fcvm.2023.1298422

**Published:** 2023-10-04

**Authors:** Maurizio Volterrani, Giuseppe Caminiti

**Affiliations:** ^1^ Department of Human Science and Promotion of Quality of Life, San Raffaele Open University, Rome, Italy; ^2^ Cardio –Pulmonary Department, IRCCS San Raffaele, Rome, Italy

**Keywords:** physical activity, exercise training, lifestyle & behaviour, cardiovascular disease, cardiovascular prevention

**Editorial on the Research Topic** Physical Activity and Exercise for the Prevention and Management of Cardiovascular Risk and Cardiovascular Disease

Sedentary lifestyle is a major cardiovascular disease (CVD) risk factor being related to a higher cardiovascular mortality and morbidity (1). Conversely, performing physical activity, mostly in the form of structured exercise training (ET) programs, has proven to be a safe and effective non-pharmacological intervention for counteracting CVDs risk factors, preventing CVDs and mitigating their clinical impact (2). However several questions, regarding how, when and how long ET should be administered, still remain unanswered. In general terms 150–300 min/week of moderate-to-intensity physical activity are strongly recommended for providing substantial health benefits and lowering cardiovascular mortality and morbidity (3). Moreover, the exact “doses” and modalities of exercise needed in different clinical contexts for reaching specific goals, remains to be established. The seven article collected in the present Research Topic contribute to fill the gap in the current knowledge, since they investigated the role of physical activity and ET in some conditions of considerable clinical relevance: from subjects with hypertension to specific groups at high risk profile such as post-menopausal women, patients with coronary artery disease (CAD), myocardial infarction (MI) and heart failure.

There is la large body of scientific evidences regarding the effects of ET, alone or in association with pharmacological therapy, in treating hypertension and in preventing arterial blood pressure (BP) increases in normotensive subjects (4). Overall a significant decrease of BP values following aerobic continuous and interval training have been wildly described. Conversely, the effects of strength training on BP are traditionally more controversial and there is a need for further studies.

In the original research article by Hidayat et al. the relationship between strength training volume and blood pressure in a population of young healthy subjects was investigated.

The authors found that intensity and duration of the strength exercise program were both directly related to systolic BP increases, particularly in male subjects with high BP values and low cardiorespiratory fitness.

These results suggest interesting considerations: firstly, when strength training is prescribed with the aim to impact BP, the prescriber should weigh up the dose of exercise in accordance to a person's premorbid conditions; secondly, the improvement of cardiopulmonary fitness through aerobic training before performing strength training, could mitigate the impact of the latter on BP values. Several researches have also investigated the effects of low-intensity exercise modalities, including oriental disciplines, on BP values. Baduanjin is one of traditional Chinese exercise of mild to moderate intensity that emphasizes the mind-body connection and that consists of slow movements while breathing deeply, and muscle stretching with mental concentration. This exercise modality has already convincing evidences of producing therapeutic benefits in people with a wilde range of chronic conditions (5). The effects of Baduanjin on BP have also been quantified (6). However previous meta-analyses were limited by low number and by high heterogeneity of studies included. In this research Topic, Ma et al. tried to overcome these limitations by publishing the largest meta-analysis testing the efficacy of Baduanjin in reducing BP. Authors analysed 12 databases and included a total of 28 studies. They found that Baduanjin, when exercised every day, produced reductions of systolic of 9.3 mmHg and reductions of diastolic BP of 6.3 mmHg. These results appear to be very promising particularly for elderly and frail hypertensive subjects who are prevented or not willing to perform exercise at higher intensities and in which Baduanjin could be taken into consideration in addition to their pharmacological therapy. Arterial stiffness is related to hypertension and it is a prognostic marker in patients with CVDs (7). Beneficial effects of ET on cardiovascular system are partly linked to its ability to improve arterial stiffness (8). In this Research Topic Huang et al. investigated the acute effects of ET on arterial stiffness in a large sample of healthy individuals. Interestingly authors divided the population in different groups according to their body fat percentage. They observed improvements in arterial stiffness all across the different groups. However, subjects with low body fat percentage showed the greatest reduction in arterial stiffness after exercise. Results of this research suggest that different doses of exercise should be administered according to body fat percentage in order to maximize its benefits on cardiovascular parameters. The study again underline the importance to take into account risk profiles and comorbidities when prescribing ET since they can affects the response to exercise. Post-menopause women experience changes in cardiometabolic, physical, and psychosocial health that negatively impacts their overall quality of life. Some of these changes such as weight gain, increases in fat mass, insulin resistance and vascular dysfunction, contribute to an increased risk of CVDs. Practicing physical activity and ET during menopause, by counteracting these factors, can potentially reduce cardiovascular risk and attenuate health declines. Kalhafi et al. performed a meta-analysis with the aim to establish the efficacy of ET in post-menopausal women. They found that exercise, aerobic, resistance, and combined exercise trainings were all effective in increased cardiorespiratory fitness. Remarkably this is the first meta-analysis investigating ET effects in this population. Future studies are needed in order to establish whether the improvement of cardiorespiratory fitness will translate into a reduction of cardiovascular risk for post-menopausal women. Cai et al. investigated the impact of having an history of habitually performing physical activity on the prognosis of patients hospitalized for acute MI. They found that patients who had an history of practicing habitual physical activity had lower rate of cardiovascular events and lower 1-year death rate for cardiovascular reasons, following MI. These results are of particular interest since they focus on a group of patients with a very high risk profile. Clearly further prospective studies are needed in order to establish what are the best volumes and modalities of exercise that confer health protection in the case of MI. Among different data derived from the ergometric test, heart rate recovery (HRR) is particularly interesting since it represents the balance between parasympathetic reactivation and sympathetic regression at the end of the exercise phase. HRR has proved to predict outcome in patients with CAD but which is the best HRR time point for predicting cardiovascular events in such patients is still unclear. The study of Yang et al. published in this Research Topic, significantly contributes to identify this point: the authors observed that HRR at the third minute had the greatest predictive power compared to HRR measured at earlier time points. This result could improve our ability to make the prognostic stratification of CAD patients. Yang et al. performed a bibliometric research providing an overall view of ET for heart failure in the past twenty years. The study showed a constant rate of publications in the western word and a growing trend of articles published in developing countries on this topic.

In conclusion, the findings of the original articles and meta-analyses of this Research Topic contribute to deepen our knowledge on the role of ET in the field of cardiovascular protection. At the same time, they move in the direction of a more individually tailored ET prescription.
